# New insights into the association between cardiometabolic index with metabolic profile, nutritional status, and inflammaging in older adults

**DOI:** 10.3389/fragi.2025.1699767

**Published:** 2026-01-12

**Authors:** Sylvia Ramuth, Rafael Leite Carvalho, Rafael Zappitelli Moscogliato, Marcelo Rossi, Luiz Henrique da Silva Nali, Patrícia Colombo-Souza, Jônatas Bussador Do Amaral, Guilherme Eustáquio Furtado, Tábatta Renata Pereira de Brito, André Luis Lacerda Bachi

**Affiliations:** 1 Post-Graduation Program in Health Sciences, Santo Amaro University (UNISA), São Paulo, Brazil; 2 Department of Otorhinolaryngology — Head and Neck Surgery, ENT Research Laboratory, Federal University of São Paulo (UNIFESP), São Paulo, Brazil; 3 Post-Graduation Program in Medicine (Otorhinolaryngology), Federal University of São Paulo (UNIFESP), São Paulo, Brazil; 4 Polytechnic Institute of Coimbra, Coimbra, Portugal; 5 SPRINT - Sport Physical Activity and Health Research & Innovation Center, Polytechnic Institute of Coimbra, Coimbra, Portugal; 6 Research Centre for Natural Resources Environment and Society (CERNAS), Polytechnic Institute of Coimbra, Coimbra, Portugal; 7 Faculty of Nutrition, Federal University of Alfenas, Alfenas, Brazil

**Keywords:** aging, cardiometabolic index, cytokines, inflammation, nutritional status

## Abstract

**Introduction:**

Cardiometabolic index (CMI) has been highlighted as a useful tool for predicting cardiovascular and metabolic disease, but its association with systemic inflammatory status in the aged population is not yet fully understood. Thus, we investigated the association between the CMI and the triad -metabolic profile X body mass index X inflammaging -in older adults classified as having or not having obesity.

**Methods:**

A total of 132 older adults of both sexes (women-68; men-64, mean age of 71.3±6.5 years), participated in this study. Demographic and anthropometric data, as well as blood samples, were collected to assess blood glucose, lipids, protein, and inflammatory profiles.

**Results:**

Initially, the volunteers were separated according to the CMI values into two groups: G1 (<50% of the mean CMI value) and G2 (>50% of the mean CMI value). Volunteers in the G2 group, regardless of gender, presented not only lower HDL-c values but also higher weight, BMI, levels of total cholesterol, LDL-c, triglycerides, and the triglycerides to HDL ratio (TG/HDL) than the G1 group. The correlation analysis and linear multivariate regression, with CMI-adjustment, showed a significant positive association with BMI, as well as with pro-inflammatory cytokines, both in the G1 and G2 groups, regardless of gender. After that, the volunteers were separated according to BMI into normal weight and those with obesity. In general, the G2 subgroups with obesity showed higher levels of pro-inflammatory cytokines IL-1β, IL-6, IFN-γ, and TNF-α than the respective G1 subgroups, and also an association of CMI in favor of a pro-inflammatory systemic status, particularly in the older women group.

**Conclusion:**

In this cross-sectional study, our findings not only reinforce the potential role of CMI in cardiovascular risk assessment but also may putatively suggest that this index has an interesting association with systemic pro-inflammatory status in older adults, preferentially with obesity.

## 1 Introduction

There is a consensus that population aging worldwide is one of the most important contemporary human issues, reflecting both socioeconomic development and the triumph of public health (World Health Organization, 2025a). This situation is achieved by the decline in the birth rate combined with the decline in mortality, resulting in a significant increase in the older population (World Health Organization, 2025b; Samir and Levin, 2013; Lee et al., 2014; Stoodley and Conroy, 2024). Corroborating with this fact, according to the World Health Organization (2025a), it is estimated that “By 2030, 1 in 6 people in the world will be aged 60 years or over. At this time the share of the population aged 60 years and over will increase from 1 billion in 2020 to 1.4 billion. By 2050, the world’s population of people aged 60 years and older will double (2.1 billion)” (World Health Organization, 2025a).

Although population aging is increasing worldwide, it is important to remember that living longer is not synonymous with living well (Stoodley and Conroy, 2024). In this sense, the incidence of chronic and degenerative diseases is highlighted as one of the main factors contributing to mortality in the older population (Culberson et al., 2023). In 2008, of the 36 million deaths worldwide, 63% were due to chronic non-communicable diseases (NCDs), particularly diabetes, cancer, cardiovascular diseases and chronic respiratory diseases (World Health Organization, 2025b; Culberson et al., 2023).

Age-related diseases are known to be influenced by various factors, including genetic, epigenetic, metabolic, immunological and inflammatory changes (Tenchov et al., 2024). Regarding specifically the effects of inflammation on aging, it has been suggested since 2000 that so-called inflammaging, a phenomenon manifested by chronic, sterile and low-grade systemic inflammation associated with aging (Franceschi et al., 2000), not only leads to significant alterations in immune/inflammatory responses but is also associated with the progression of the aforementioned age-related diseases (Franceschi et al., 2000; Huang et al., 2025).

Additionally, the increase in systemic concentrations of pro-inflammatory cytokines, such as tumor necrosis factor alpha (TNF-α) and interleukin (IL)-6, in older adults are due to the increased accumulation of body fat, especially in the central, abdominal and visceral areas of the body (Singh and Newman, 2011). According to the literature, obesity in the older population has increased proportionally on all continents over the last 30 years (Franceschi et al., 2007; Malenfant and Batsis, 2019). It is therefore evident that the increase in obesity, which not only leads to significant metabolic changes but is also associated with an increase in pro-inflammatory cytokines, favors both the development and progression of inflammaging and also of metaflammation, a term coined in 2018 to define the systemic inflammatory state of metabolic origin (Teissier et al., 2022; Hotamisligil, 2017; Franceschi et al., 2018).

Although metaflammation increases the risk of developing chronic diseases, particularly metabolic diseases (Teissier et al., 2022), there is still debate about the need to determine biomarkers capable of more accurately reflecting the systemic and chronic inflammatory state and thus predicting the prognosis of clinical conditions associated with aging, preferably those related to metabolism. Therefore, the cardiometabolic index (CMI) has recently emerged as a useful and promising tool, as it combines important metabolic parameters such as triglycerides and HDL cholesterol with body parameters such as height and waist circumference. It is worth mentioning that it was reported that measures of central obesity, such as Waist-to-Hip Ratio (WHR) and Waist-to-Height Ratio (WHtR), generally predict cardiometabolic outcomes more reliably than BMI alone (Lee et al., 2008). Even CMI uses the Waist-to-Height Ratio (WHtR), its ability to predict cardiovascular risk, especially in situations where metabolic dysfunction and chronic inflammation coexist, is still being investigated (Kolb, 2022; Tang et al., 2024). Indeed, individuals with an altered cardiometabolic profile often present insulin resistance and metabolic syndrome, arterial hypertension, chronic systemic inflammation and accumulation of abdominal visceral fat, which increases the secretion of pro-inflammatory cytokines (Oliveira et al., 2010; Banerjee and Mani, 2025).

It is also worth mentioning that even though the metabolic and inflammatory alterations are seen in the older adults can occur in both sexes, it is known that there are significant differences between them, which is related to some factors such as genetic, hormonal, environmental and immunosenescence (Carvalho et al., 2024; Baker et al., 2025). In this sense, it was reported that men and women show different cardiometabolic risk factors, e.g., while women tend to accumulate more subcutaneous fat, men have a greater tendency to accumulate visceral fat, which is more associated with cardiovascular diseases and insulin resistance (Baker et al., 2025; Rohden, 2008). Among the aspects associated with immunosenescence and its impact on cardiometabolic risk, it should be noted that women generally have a more robust immune response throughout life, but the higher immune activation may lead to a greater predisposition to autoimmune diseases and increased inflammatory processes associated with senescence. In contrast, men tend to have a less efficient immune response as they age, which may contribute to a greater susceptibility to infection and a decreased ability to respond to inflammatory insults (Calabrò et al., 2023).

Although CMI has been shown to be useful for cardiovascular and metabolic disease risk prediction, the proposition that this index may serve as an indicator of other situations related to the presence of inflammaging and/or metainflammation in older people has been poorly understood. Therefore, in this study, we aimed to investigate the association between CMI and the triad - metabolic profile X body mass index X inflammaging - in older women and older men classified as having or not obesity.

## 2 Materials and methods

### 2.1 Design of the study

In this retrospective cross-sectional and observational study, which followed the STROBE (Strengthening the Reporting of Observational Studies in Epidemiology) guideline to ensure methodological rigor, 132 older adults of both sexes (68 women and 64 men) aged between 60 and 90 years were enrolled. In addition, the participants were separated into two groups based on CMI scores: group G1 with subjects whose CMI values were 50% below the determined average, and group G2 with subjects whose CMI values were 50% above the determined average.

### 2.2 Data and biological samples of participants

The present study was developed with data and serum samples kindly provided by Prof. Dr. Tábatta Renata Pereira de Brito, Professor at the Faculty of Nutrition of the Universidade Federal de Alfenas (UFAL), Alfenas, Brazil. Both the data and the biological samples were collected in the first 3 months of 2019, i.e., before the COVID-19 pandemic. All volunteers came from the city of Alfenas, which is located in the south of the state of Minas Gerais.

### 2.3 Recruitment of volunteers

Initially, a total of 250 older adults’ residents of Alfenas city were invited to participate in Prof. Dr. Tábatta Renata Pereira de Brito’s research project. Selection criteria were used to exclude 118 volunteers, thus resulting in 132 participants who were included in the present study.

### 2.4 Selection criteria

The inclusion criteria were: (i) age between 60 and 90 years at the time of recruitment; (ii) no symptoms of disease at the time of data collection and collection of biological samples; (iii) no HIV seropositive; (iv) no neurological, chronic kidney and/or liver disease; (v) no cancer; (vi) and consent to participate in the study. The exclusion criteria were: (i) corticosteroid therapy or use of any other non-steroidal anti-inflammatory drug for a period of up to 2 months prior to the collection of data and biological samples; (ii) and non-participation in any phase of the study.

### 2.5 Sample size calculation and experimental groups

To develop this study, a sample size calculation was performed to determine a minimum number of participants using the program G*Power, version 3. On this basis, the sample size and statistical power were estimated based on the effect size of 0.30, the α-level of 0.05 (5%), the statistical power of 0.95 and a sample loss of 10%. The calculation showed that 124 participants would be sufficient to conduct this study.

### 2.6 Ethical aspects

After all the procedures of the study were presented and explained in detail to the volunteers, those who agreed to participate gave their consent by signing the Informed Consent Form, previously approved by the Ethics and Research Committee of the Universidade Federal de Alfenas (UFAL), under number 2,668,936. This study was conducted following Declaration of Helsinki (Petrini, 2014) and also guidelines established in Resolution No. 466/2012 of the National Health Council (Brasil. Ministério da Saúde, 2013).

### 2.7 Clinical, physical and anthropometric assessment

Data on clinical and physical status were collected at the volunteers' first visit to UFAL, with information obtained through self-reporting by the participants. In addition, anthropometric measurements were performed using a standard stadiometer (height in cm) and a digital scale (weight in kg) according to the standard protocol, in which participants were instructed to wear light clothing and be barefoot. The body mass index (BMI) was calculated based on the weight and height values and was used to classify the individuals as underweight, eutrophic (normal), overweight and with obesity, according to the criteria proposed by the WHO, as previously described (Huang et al., 2025). Additionally, waist circumference was measured using a non-elastic tape measure. In this sense, participants were instructed to stand with their feet together, ensuring that their weight was evenly distributed between their legs and that their arms were relaxed at their sides. Then, the tape measure was placed horizontally around the abdomen, midway between the lowest rib and the iliac crest, in order to avoid compressing the skin and allowing clothing to interfere with the accuracy of the measurement. It is worth noting that these procedures were performed in a private setting to ensure the full comfort and privacy of the participants.

### 2.8 Serum sample collection

Peripheral fasting blood samples were collected in appropriate tubes to obtain serum aliquots (at least 500 μL), from blood clotting in the collection tube itself and after centrifugation at 800 × g for 10 min at 4 °C. The serum aliquots obtained were frozen at −80 °C until they were used for the biochemical/inflammatory analysis as described below.

### 2.9 Assessment of biochemical parameters

Serum concentrations of glucose, total cholesterol and fractions (HDL cholesterol and LDL cholesterol), triglycerides, and total proteins were determined in previously stored serum samples using commercially available colorimetric kits (Bioclin, Brazil) according to the manufacturer’s instructions.

### 2.10 Assessment of systemic cytokine concentrations

Serum concentrations of the cytokines IL-1β, IL-6, IL-10, IFN-γ and TNF-α were measured using the LEGENDplex™ bead-based multiplex assay (Biolegend, San Diego, CA, United States) following the manufacturer’s recommendations and instructions. Each cytokine concentration was calculated using the respective standard curve. In addition, the linearity of the multiplex assay ranged from 2.4 to 10,000 pg/mL, with correlation coefficients of all standard curves between 0.95 and 0.99. The intra-assay coefficients of variance were 2–5% and the inter-assay coefficients of variance were 7%–9%. All analyses were performed on a BD Accuri™ C6 Plus flow cytometer (BD Biosciences, San Jose, CA, United States) and the data obtained were analyzed using LEGENDPlex™ V8.0 software (Biolegend, San Diego, CA, United States).

### 2.11 Statistical analysis

First, the Shapiro-Wilk and Levene tests were used to assess both the normality of the data and the homogeneity of variances. On this basis, the parametric variables (demographic, anthropometric and biochemical data) were presented as mean values and standard deviation (X_±_SD) and compared using the Student's t-test and one-way ANOVA with Tukey *post hoc* test, while the non-parametric variables (inflammatory cytokines) were presented as median and interquartile ranges (X_IR) and compared using the Mann-Whitney test and Kruskal-Wallis test with Müller-Dunn posttest. In addition, Pearson (parametric variables) or Spearman (non-parametric variables) correlation coefficient tests, as well as a multivariate linear regression analysis with adjustment for cardiometabolic index (CMI) values, were used. All analyses were performed using GraphPad Prism 8.1.2 software, and a p-value of 5% (p ≤ 0.05) was set.

Additionally, another multiple linear regression, but using the backward selection method, was performed in the volunteer groups defined by the combination of sex, nutritional status, and stratification by the Cardiometabolic Index (CMI). In all models, CMI was the dependent variable, and the cytokines IL-1β, IL-6, IL-10, IFN-γ, and TNF-α were initially included as predictors. All analyses were performed using Python (version 3.11). Data manipulation and preprocessing were conducted with pandas (v2.1.4) and numpy (v1.26.2). Multiple linear regression models were fitted using the Ordinary Least Squares (OLS) method implemented in the statsmodels’ package (v0.14.1). The backward stepwise selection procedure was applied to identify the most parsimonious models by sequentially removing variables with the highest p-value until only predictors with p < 0.15 remained in the final model. This criterion was adopted to account for the exploratory nature of the analysis and the limited sample size, as suggested in regression modeling literature (Draper and Smith, 1998). The initial models included the cytokines IL-1β, IL-6, IL-10, IFN-γ, and TNF-α as independent variables, with the Cardiometabolic Index (CMI) as the dependent variable. For each backward iteration, the following parameters were recorded: β coefficients with 95% confidence intervals (CI), p-values, and coefficients of determination (R^2^ and adjusted R^2^). For the final model in each subgroup, the F-statistic and its associated p-value were reported to assess overall model significance. Volunteer groups were analyzed separately according to sex (male/female), nutritional status (eutrophic/obese), and CMI stratification (low/high based on the median within each stratum).

In order to summarize redundant information across the pro-inflammatory cytokine panel assessed here and capture their shared variance structure, a Principal Component Analysis (PCA) was performed using IL-1β, IL-6, TNF-α, and IFN-γ. The first principal component (PC1), which accounted for the largest proportion of covariance among these mediators, was extracted and used as a synthetic marker of inflammatory load. This axis was defined as the Pro-inflammatory Gradient Score (PGS), which has higher values indicating a greater overall pro-inflammatory burden. Then, the PGS was used as a continuous variable in subsequent analyses to characterize the inflammatory gradient across individuals and explore how this gradient relates to cardiometabolic status.

Besides, to verify whether the median-based stratification of the CMI reflected a biologically meaningful separation among volunteers with distinct risk profiles, a Receiver Operating Characteristic (ROC) analysis was conducted. It is worth mentioning that ROC curves were generated separately for older men and older women, using obesity status (yes/no) as the binary outcome and CMI as the predictor. In parallel, we evaluated the discriminative ability of the Pro-inflammatory Gradient Score (PGS), derived from the PCA of the cytokine matrix, to classify individuals with higher cardiometabolic burden (upper quartiles, Q3–Q4, versus lower quartiles, Q1–Q2). All ROC analyses were performed in R using the pROC package, and area under the curve (AUC) values with 95% confidence intervals were computed to quantify discriminative ability.

In a complementary analysis, it was specifically evaluated whether IL-10 modified the association between pro-inflammatory burden and CMI by fitting linear regression models with an interaction term between the Pro-inflammatory Gradient Score (PGS) and IL-10. For these models, CMI was the dependent variable, and z-standardized PGS (derived as the first principal component of IL-1β, IL-6, TNF-α, and IFN-γ) and IL-10 were entered as continuous predictors along with their product term (z-PGS × z-IL-10) to capture effect modification. Interaction models were first fitted in the overall sample and then stratified by sex, and we extracted β-coefficients, 95% confidence intervals, p-values, and coefficients of determination (R^2^). To aid interpretation, predicted CMI values across the z-PGS range at representative low and high IL-10 levels (defined by the sample media). All data and results were tabulated and exported directly within the R environment. Regression models, PCA-derived scores, and interaction analyses (CMI ∼ PGS × IL-10) were conducted in R version 4.3.3 using RStudio 2025.09.2 Build 418. Data preprocessing and manipulation were performed with the readxl and dplyr packages, while PCA computation and score extraction (PGS) were implemented with base R functions. All figures associated with the regression and interaction models were generated using ggplot2 and exported with ggsave, ensuring full reproducibility of the analytical workflow.

## 3 Results

Table 1 presents the data (mean and standard deviation) on the demographic data (age), anthropometric data (weight, height and BMI), cardiometabolic index (CMI), biochemical profile (glycemia, lipid profile and total proteins), clinical (type 2 diabetes mellitus, arterial hypertension, and cardiovascular diseases), and lifestyle habits (physical activity practice and current smoking) of the older volunteers who participated in this study, both overall and separately by male and female gender. It was found that the older men group had higher weight and height values than the older women group. No other significant differences were found.

**TABLE 1:** Data of age (demographic), anthropometric, and biochemical (lipid profile, glucose and total proteins concentration), presented as mean and standard deviation (X±SD) as well as clinical and lifestyle habits (presented as number of volunteers), of the volunteers participating in this study, both overall and separated by sex (older women and older men).

Parameters	Volunteers			*p*-value^a^
	Total (n = 132)	Women (n = 68)	Men (n = 64)	
Age (years)	71.3 ± 6.5	70.9 ± 5.6	71.7 ± 7.4	0,5495
Weight (kg)	72.35 ± 17.86	66.87 ± 16.23^a^	78.44 ± 18.05	**0,0002**
Height (cm)	161.2 ± 9.90	154.9 ± 8.1^a^	167.9 ± 7.0	**<0,0001**
BMI (kg/m^2^)	27.79 ± 6.10	27.88 ± 6.31	27.78 ± 5.90	0,9268
Glucose (mg/dL)	105.5 ± 33.73	103.1 ± 33.53	107.9 ± 34.03	0,4232
Total cholesterol (mg/dL)	193.8 ± 53.63	201.2 ± 55.74	186.0 ± 50.63	0,1038
HDL-cholesterol (mg/dL)	43.53 ± 8.30	43.35 ± 7.81	43.72 ± 8.82	0,7985
LDL-cholesterol (mg/dL)	113.4 ± 49.04	120.2 ± 52.17	107.7 ± 42.56	0,1356
Triglycerides (mg/dL)	184.2 ± 51.95	188.4 ± 52.60	179.8 ± 51.31	0,3447
TG/HDL ratio	6.86 ± 1.69	4.5 ± 1.6	4.3 ± 1.4	0,4095
Total protein (g/L)	4.4 ± 1.5	6.98 ± 1.62	6.75 ± 1.77	0,5324
CMI	2.8 ± 1.3	2.9 ± 1.3	2.7 ± 1.2	0,3078
Clinical (n)^b^				
Type 2 diabetes mellitus	48	20	28	0.0870
Arterial hypertension	90	42	48	0.1028
Cardiovascular disease	38	17	21	0.3218
Lifestyle (n)^a^				
Physical activity practice	41	20	21	0.6730
Current smoker	17	8	9	0.6937

^a^ Comparison between the values obtained in the older women and older men groups.

^b^ n = number of individuals.

BMI, body mass index; HDL, high-density lipoprotein; LDL, low-density lipoprotein; TG/HDL, Triglycerides and HDL-cholesterol; CMI, cardiometabolic index.

In bold are the values statistically significant.

At this point, it is important to mention that the results found in the ROC analyses (Supplementary Figure S1) showed that CMI displayed excellent discriminatory ability for identifying obesity in both older men and older women volunteers, thus reinforcing the separation of volunteers into two groups based on CMI values. Of note, in both sexes, AUC values were high (AUC >0.97), with narrow confidence intervals, indicating outstanding sensitivity and specificity. Additionally, the evaluation of the pro-inflammatory PCA-derived axis (PC1_high_Q3-Q4) also demonstrated meaningful discriminatory performance for identifying individuals with higher cardiometabolic burden. AUC values were 0.86 in the older men subgroup and 0.90 in the older women subgroup, also reinforcing the good classification accuracy. Although these values were lower than those observed for CMI against obesity, they indicate that the inflammatory gradient captured by PC1 retains substantial information related to cardiometabolic risk. Taken together, these analyses show that median-based stratification of CMI does not create artificial groups but instead reflects physiologically coherent distinctions in cardiometabolic status, thus supporting the simultaneous use of CMI as a continuous variable in inferential models and as a categorical variable for descriptive and exploratory analyses. Therefore, the following results show the data obtained when the volunteers were initially separated into two groups based on CMI values and subsequently separated using the BMI values.

In Table 2 is shown the data concerning demographic (age), anthropometric (weight, height, and BMI), cardiometabolic index (CMI), biochemical profile (glycemia, lipid profile, and total proteins), and clinical (type 2 diabetes mellitus, arterial hypertension, and cardiovascular diseases) of volunteers not only separated into older men and older women groups, but also according to their CMI values, i.e., G1 group (>50% of the mean value) and G2 group (<50% of the mean value). It is worth noting that, similarly to what was observed in Table 1, in the intergroup analysis, the older men group presented higher weight and height values than the values found in the older women group, regardless of the CMI separation. In addition, in the intragroup analysis, the volunteers in G2 groups, both older women and older men, showed not only lower HDL-cholesterol values, but also higher weight, BMI, total cholesterol, LDL-cholesterol, triglycerides, TG/HDL ratio values, as well as increased number of volunteers who had arterial hypertension and cardiovascular diseases than the values found in G1 groups. No differences were found in the number of volunteers who reported practicing physical activity and were current smokers.

**TABLE 2:** Demographic, anthropometric and biochemical data (lipid profile, glucose and total proteins concentration), presented as mean and standard deviation (X±SD), as well as clinical and lifestyle habits (presented as number of volunteers), of the volunteers participating in this study, separated by sex (older women and older men). and CMI values.

Parameters	Volunteers						
	Women G1 (n = 34)	Women G2 (n = 34)	*p*-value (intragroup)	Men G1 (n = 32)	Men G2 (n = 32)	*p*-values (intragroup)	*p*-value (intergroup)
Age (years)	71.4 ± 4.8	70.3 ± 6.4	0,4333	71.8 ± 6.8	71.8 ± 7.9	0,9944	0,7827/0,4197
Weight (kg)	54.72 ± 7.80	80.17 ± 12.03	**<0,0001**	64.40 ± 6.50	93.35 ± 13.88	**<0,0001**	**<0,0001** **/** **0,0001**
Height (cm)	156.2 ± 8.9	153.4 ± 6.9	0,3162	168.8 ± 6.4	167.0 ± 7.5	0,1650	**<0,0001** **/** **<0,0001**
BMI (kg/m^2^)	22.58 ± 1.54	33.31 ± 3.02	**<0,0001**	22.37 ± 1.96	33.91 ± 2.95	**<0,0001**	0,6241/0,4282
Glucose (mg/dL)	104.73 ± 6.62	111.3 ± 31.19	0,4380	105.9 ± 34.26	100.1 ± 32.98	0,4818	0,8875/0,1703
Total cholesterol (mg/dL)	186.0 ± 46.72	215.1 ± 60.21	**0,0314**	172.0 ± 33.87	198.9 ± 59.78	**0,0318**	0,2642/0,1776
HDL-cholesterol (mg/dL)	48.43 ± 7.7	37.79 ± 1.64	**<0,0001**	48.52 ± 9.70	38.46 ± 2.81	**<0,0001**	0,9663/0,2552
LDL-cholesterol (mg/dL)	105.0 ± 42.31	134.1 ± 56.89	**0,0218**	97.11 ± 28.68	117.4 ± 50.63	**0,0493**	0,2044/0,3883
Triglycerides (mg/dL)	163.2 ± 44.41	216.0 ± 47.16	**<0,0001**	164.6 ± 48.84	196.6 ± 49.39	**0,0109**	0,9030/0,1155
TG/HDL ratio	3.4 ± 0.89	5.7 ± 1.2	**<0,0001**	3.4 ± 0.7	5.3 ± 1.4	**<0,0001**	0,9651/0,1682
Total protein (g/L)	6.92 ± 1.69	7.58 ± 0.67	0,4431	6.73 ± 1.57	6.81 ± 2.43	0,9062	0,6417/0,5512
Clinical (n)^a^							
Type 2 diabetes mellitus	7	13	0.1113	12	18	0.1329	0.1295/0.1428
Arterial hypertension	17	25	**0.0459**	20	28	**0.0209**	0.3065/0.1538
Cardiovascular disease	5	12	**0.0499**	6	15	**0.0166**	0.6595/0.3389
Lifestyle (n)*							
Physical activity practice	7	11	0.1684	7	14	0.0624	0.8983/0.3401
Current smoker	5	4	0.7192	4	4	0.9999	0.6479/0.9271

^a^ n = number of individuals.

BMI, body mass index; HDL, high-density lipoprotein; LDL, low-density lipoprotein; TG/HDL, Triglycerides and HDL-cholesterol; CMI, cardiometabolic index. Red values present the p-values obtained in the comparison between women’s groups (G1 × G2 groups). Blue values present the p-values obtained in the comparison between men´s groups (G1 × G2 groups).

In bold are the values statistically significant.

**FIGURE 1:**
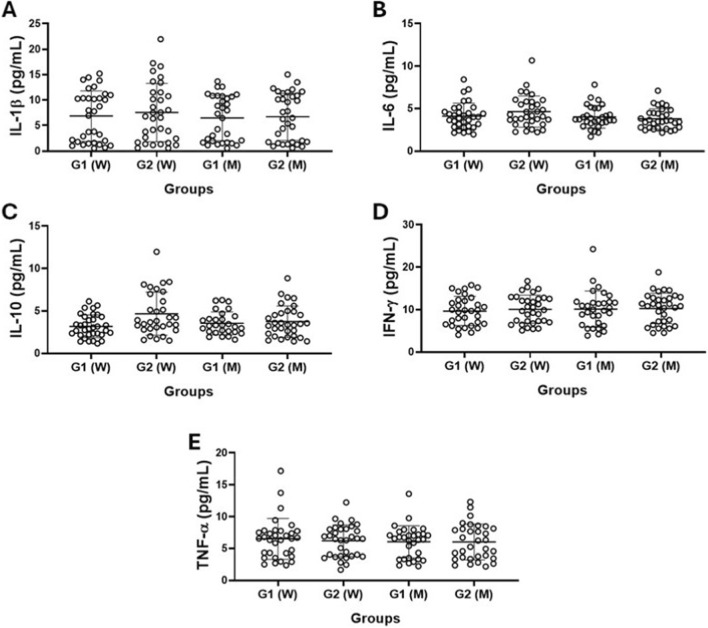
Serum levels of cytokines IL-1β **(A)**, IL-6 **(B)**, IL-10 **(C)**, IFN-у **(D)**, and TNF-α **(E)** in older women (W) and older men (M) groups separated according to CMI values into G1 (>50% of CMI values) and G2 (<50% of CMI values) groups. Values are presented as median and interquartile range.

Figure 1 shows the results obtained in the evaluation of serum levels of the cytokines IL-1β (Figure 1A), IL-6 (Figure 1B), IL-10 (Figure 1C), IFN-γ (Figure 1D), and TNF-α (Figure 1E) in the older women (W) and older men (M) groups separated into G1 and G2 groups. No significant difference was evidenced in the volunteer groups.

**FIGURE 2:**
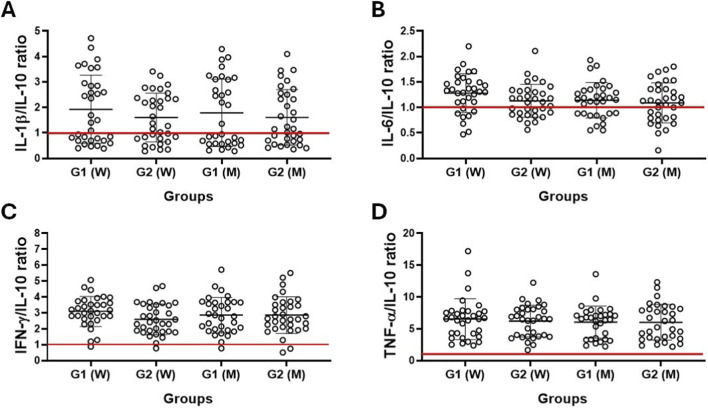
Ratio between serum levels of pro- and anti-inflammatory cytokines [IL-1β/IL-10 **(A)**, IL-6/IL-10 **(B)**, IFN-у/IL-10 **(C)**, and TNF-α/IL-10 **(D)**] in older women (W) and older men (M) groups separated according to CMI values into G1 (>50% of CMI values) and G2 (<50% of CMI values) groups. Values are presented as median and interquartile range. The red line at number 1 represents equality of concentrations.

Figure 2 shows the results of the evaluation of the ratio between the serum levels of the pro-inflammatory cytokines IL-1β (Figure 2A), IL-6 (Figure 2B), IFN-γ (Figure 2C) and TNF-α (Figure 2D) and the anti-inflammatory cytokine IL-10 in the older women (W) and older men (M) groups separated into G1 and G2 groups. Again, no significant difference was found between the volunteer groups.

Table 3 shows the results obtained in the Spearman´s correlation coefficient analysis, with CMI values as the main variable, in the participating volunteers (older men and older women) separated into G1 and G2 groups. It can be observed that, regardless of the separation into G1 and G2 groups, significant positive correlations were found between the CMI and BMI, as well as between CMI and cytokines of both pro- and anti-inflammatory profiles in the group of older men and older women volunteers.

**TABLE 3:** The Spearman correlation coefficient analysis highlights the significant relationships found between CMI as the main variable and the other parameters (BMI and inflammatory cytokines) in the volunteer groups (older men and older women) separated according to the values found for CMI (G1 = <50% values; and G2 = >50% values).

G1 group – older men			G2 group – older men		
Parameters	*rho*-value	*p*-value	Parameters	*rho*-value	*p*-value
CMI × BMI	0,7846	<0,0001	CMI × BMI	0,7667	<0,0001
CMI × IL-1β	0,6249	0,0001	CMI × IL-1β	0,7630	<0,0001
CMI × IL-1β/IL-10	0,7461	<0,0001	CMI × IL-6	0,4615	0,0068
CMI × IFN-γ	0,3766	0,0367	CMI × IL-6/IL-10	0,4976	0,0032
CMI × IFN-γ/IL-10	0,4854	0,0056	CMI × IL-10	0,6418	<0,0001
CMI × TNF-α/IL-10	0,5116	0,0032	CMI × TNF-α	0,6975	<0,0001
CMI × BMI	0,7400	<0,0001	CMI × IFN-γ	0,3766	0,0367
CMI × IL-1β	0,7126	<0,0001	CMI × BMI	0,7205	<0,0001
CMI × IL-1β/IL-10	0,5829	0,0002	CMI × IL-1β	0,7109	<0,0001
CMI × IL-6	0,4946	0,0025	CMI × IL-1β/IL-10	0,6616	<0,0001
CMI × IL-10	0,4378	0,0086	CMI × IL-6	0,4048	0,0238
CMI × IFN-γ	0,5417	0,0007	CMI × IFN-γ	0,7385	<0,0001
CMI × TNF-α	0,5591	0,0004	CMI × TNF-α	0,6738	<0,0001

Table 4 shows the results of the multivariate linear regression analysis with adjustment for CMI values in the participating volunteers (older men and older women), who were separated into G1 and G2 groups. It was found that BMI had a significant association on CMI, regardless of these volunteer groups. Interestingly, pro-inflammatory systemic status showed a significant influence on CMI in the G1 groups (both in older men and older women). On the other hand, a single significant association of TNF-α/IL-10 ratio in CMI was observed in the G2 older women group.

**TABLE 4:** Multivariate linear regression analysis with adjustment for cardiometabolic index (CMI) values in the volunteer groups (older men and older women), separately according to the values found for CMI (G1 = <50% values; and G2 = >50% values).

CMI-adjusted								
Parameters	G1 group – older men				G2 group – older men			
	*β−*value	CI^a^ - 95%	*p*-value	*R* ^*2​*^	β−value	CI^a^ - 95%	*p*-value	R^2^
BMI	0,0478	0.012 a 0.083	0.014	0,9396	0,0982	0.023 a 0.173	0.014	0,7656
IL-6	0,2058	0.059 a 0.352	0.012	0,9843	​	​	​	​
IL-10	−0,2767	−0.438 a −0.114	0.004	0,9947	​	​	​	​
IFN-γ	0,2473	0.142 a 0.352	0.001	0,9987	​	​	​	​
IFN-γ/IL-10	0,5845	0.289 a 0.883	0.002	0,9810	​	​	​	​
TNF-α	0,2286	0.120 a 0.337	0.001	0,9980	​	​	​	​
IL-1β/IL-10	0,2855	0.016 a 0.554	0.039	0,9782	​	​	​	​
BMI	0,0224	0.003 a 0.048	0.049	0,8614	0,2547	0.152 a 0.378	0,0001	0,9159
IL-1β	0,3128	0.054 a 0.571	0.021	0,9843	​	​	​	​
IL-1β/IL-10	1,102	0.224 a 1,981	0.017	0,9520	​	​	​	​
TNF-α	0,0151	0.005 a 0.024	0.006	0,9247	​	​	​	​
TNF-α/IL-10	0,0444	0.019 a 0,0691	0.001	0,8955	0,02371	0.001 a 0.046	0.042	0,9662
IL-6/IL-10	0,8009	0.008 a 1,593	0.047	0,9595	​	​	​	​

^a^ CI, confidence interval.

Based on the observation that BMI showed not only a close positive association but also a prominent association on CMI, the following results were obtained when the volunteer groups were again separated according to their BMI values. In this sense, it is worth noting that the BMI data allowed us to categorize the volunteers into eutrophic (normal) and with obesity subgroups.

**FIGURE 3:**
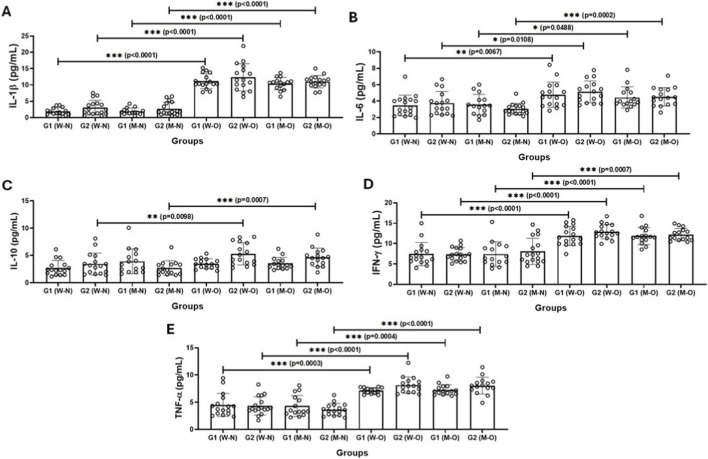
Serum levels of cytokines IL-1β **(A)**, IL-6 **(B)**, IL-10 **(C)**, IFN-у **(D)**, and TNF-α **(E)** in older women (W) and older men (M) groups separated according to CMI values into G1 (>50% of CMI values) and G2 (<50% of CMI values) groups, as well as by BMI classification into normal weight (W-N or M-W) and with obesity (W-O and M-O). Values are presented as median and interquartile range. *denotes p < 0.05; ***denotes p < 0.001.

Figure 3 shows the results of the evaluation of serum levels of the cytokines IL-1β (Figure 3A), IL-6 (Figure 3B), IL-10 (Figure 3C), IFN-γ (Figure 3D) and TNF-α (Figure 3E) in the participating volunteers (older men and older women), who were separated not only into G1 and G2 groups, but also into normal weight (N) and with obesity (O) according to BMI values. Higher IL-1β, IL-6, IFN-γ and TNF-α levels were observed in the subgroups with obesity than in the normal weight subgroups, regardless of gender. In addition, serum levels of IL-10 were higher in the G2 group (older women and older men) with obesity than in the G2 group (older women and older men) with normal weight.

**FIGURE 4:**
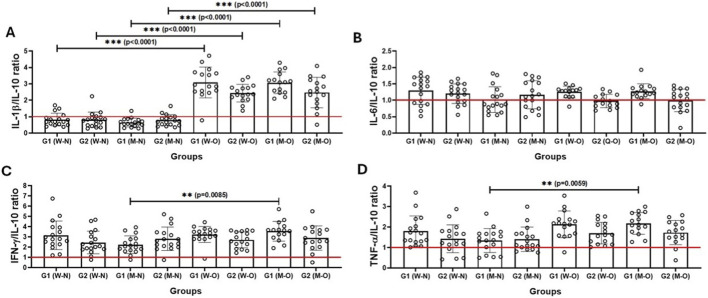
Ratio between serum levels of pro- and anti-inflammatory cytokines [IL-1β/IL-10 **(A)**, IL-6/IL-10 **(B)**, IFN-у/IL-10 **(C)**, and TNF-α/IL-10 **(D)**] in older women (W) and older men (M) groups separated according to CMI values into G1 (>50% of CMI values) and G2 (<50% of CMI values) groups, as well as by BMI classification into normal weight (W-N or M-W) and with obesity (W-O and M-O). Values are presented as median and interquartile range. *denotes p < 0.05; **denotes p < 0.01; ***denotes p < 0.001. The red line at number 1 represents equality of concentrations.

Figure 4 shows the results of the analysis of the ratio between the serum levels of the pro-inflammatory cytokines IL-1β (Figure 4A), IL-6 (Figure 4B), IFN-γ (Figure 4C) and TNF-α (Figure 4D) and the anti-inflammatory cytokine IL-10 in the participating volunteers (older men and older women), who were separated not only into G1 and G2 groups, but also according to BMI values in normal weight (N) and with obesity (O). Higher levels of the IL-1β/IL-10 ratio were observed in the subgroups with obesity than in the normal weight subgroups, regardless of gender. In addition, higher levels of IFN-γ/IL-10 and TNF-α/IL-10 ratios were found in the G2 group (older men) with obesity than in the G1 group (older men) with normal weight. No significant difference was found with regard to the IL-6/IL-10 ratio.

Table 5 shows the results of the Spearman’ correlation coefficient analysis with the CMI values as the main variable in the participating volunteers (older men and older women), who were separated not only into G1 and G2 groups, but also according to their BMI values at normal weight and with obesity. It was observed that the G2 groups with obesity had significant positive correlations between CMI and cytokines and ratios with pro-inflammatory properties, in contrast to the significant negative correlation between CMI and the IL-1β/IL-10 ratio observed in the G1 group (older women) with normal weight.

**TABLE 5:** Spearman correlation coefficient analysis highlighting the significant associations found between CMI as the main variable and inflammatory cytokines in the volunteer groups (older men and older women), separately according to the values found for CMI (G1 = <50% values; and G2 = >50% values), as well as with their BMI values at normal (appropriate) weight and with obesity.

G1 group – older women with normal weight		
Parameters	*rho*-value	*p*-value
CMI x IL-1β/IL-10	−0,6396	0,0043
G2 group – older women with obesity		
Parameters	*rho*-value	*p*-value
CMI x IL-1β	0,6418	0,0095
CMI x IFN-γ	0,6632	0,0041
CMI x IL-6/IL-10	0,4978	0,0477
G2 group – older men with obesity		
Parameters	*rho*-value	*p*-value
CM x TNF-α	0,5625	0,0291
CMI x TNF-α/IL-10	0,4803	0,0491

In Table 6 is shown the data from the linear multivariate regression analysis with adjustment for the CMI values of the participating volunteers (older men and older women), who were not only separated into G1 and G2 groups, but also according to their BMI values in normal weight and with obesity. The results showed that the proinflammatory cytokines IFN-γ and TNF-α had a significant association on CMI specifically in the older men with obesity of the G2 group, In addition, in the older women with obesity of G2 group, age showed an association with CMI.

**TABLE 6:** Multivariate linear regression analysis with adjustment for cardiometabolic index (CMI) values in the volunteer groups (older men and older women), separately according to the values found for CMI (G1 = <50% values; and G2 = >50% values), and with their BMI values at normal (adequate) weight and with obesity.

Parameters	CMi-adjusted			
	G2 group – older men with obesity			
	*β−value*	CI^a^ - 95%	*p*-value	*R* ^*2*^
IFN-γ	0,4945	0.164 a 0.823	0,0118	0,7199
TNF-α	0,1310	0.037 a 0.224	0,0153	0,1504

Parameters	G2 group – older women with normal weight			
	*β−value*	CI^a^ - 95%	*p*-value	*R* ^*2*^
Age (years)	0,0681	0.003 a 0.132	0,0419	0,6422

^a^ CI, confidence interval.

In addition to these results, we also investigated the effects of comorbidities and lifestyle habits as potential confounding factors. A multiple linear regression model adjusted for CMI or cytokines with Type 2 diabetes, arterial hypertension, cardiovascular disease, physical activity practice, and current smoking, as independent variables, was applied. Based on these data, none of the comorbidities or even lifestyle habits showed a significant association with CMI or cytokines assessed here, for each volunteer group (Supplementary Material).

Furthermore, the multiple linear regression using the backward selection method was performed in volunteer subgroups defined by the combination of sex, nutritional status, and stratification by the Cardiometabolic Index (CMI). In all models, CMI was the dependent variable, and the cytokines IL-1β, IL-6, IL-10, IFN-γ, and TNF-α were initially included as predictors. It is important to mention that application of the backward method led to the sequential removal of cytokines with the highest p-value, resulting in final models with one or two variables (see Supplementary Material). In some subgroups, a reduction in R^2^ was observed throughout the process, indicating weak contribution from the excluded cytokines.

• Older men with adequate weight:
  • G1 (<50% of CMI values): IL-6 (β = −0.1333, p = 0.0215) and IL-10 (β = 0.0836, p = 0.0245) remained in the final model (R^2^ = 0.325, F = 3.369).
  • G2 (>50% of CMI values): IL-6 (β = −0.3601, p = 0.0016) and IL-10 (β = 0.1547, p = 0.0019) were retained (R^2^ = 0.524, F = 7.711).
• Older men with obesity:
  • G1 (<50% of CMI values): IL-6 (β = 0.0904, p = 0.2752) and TNF-α (β = −0.0537, p = 0.6243) (R^2^ = 0.111, F = 0.746).
  • G2 (>50% of CMI values): IFN-γ (β = 0.207, p = 0.026) and TNF-α (β = 0.1309, p = 0.0072) (R^2^ = 0.555, F = 7.489).
• Older women with adequate weight:
  • G1 (<50% of CMI values): IL-1β (β = −0.1436, p = 0.0727) and TNF-α (β = 0.111, p = 0.0838) (R^2^ = 0.202, F = 1.899).
  • G2 (>50% of CMI values): IL-1β (β = −0.0316, p = 0.5932) and IFN-γ (β = 0.0774, p = 0.3479) (R^2^ = 0.065, F = 0.491).
• Older women with obesity:
  • G1 (<50% of CMI values): IL-1β (β = 0.0399, p = 0.3959) and IFN-γ (β = 0.0623, p = 0.1727) (R^2^ = 0.429, F = 4.882).
  • G2 (>50% of CMI values): IL-6 (β = 0.1893, p = 0.1757) and IFN-γ (β = −0.1796, p = 0.0892) (R^2^ = 0.235, F = 1.997).

**FIGURE 5:**
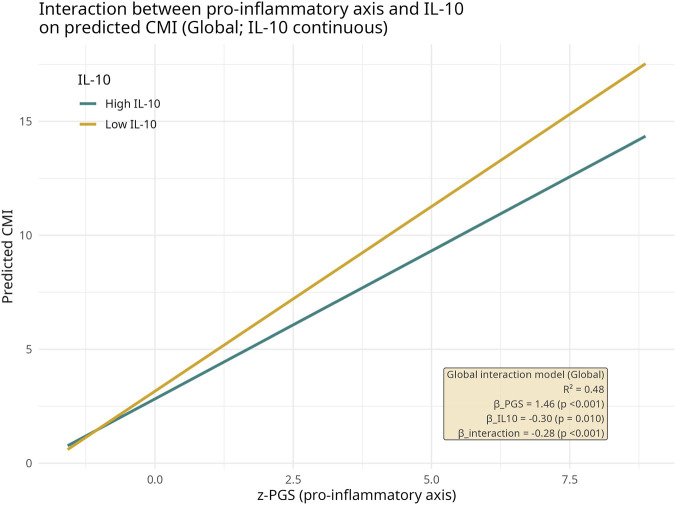
Interaction between the Pro-inflammatory Gradient Score (PGS) and IL-10 in predicting cardiometabolic status. Predicted CMI values from the global linear regression model (CMI ∼ z-PGS × z-IL-10) were plotted across the standardized pro-inflammatory axis (z-PGS). Separate lines depict model-based predictions for individuals with low and high IL-10 levels (below vs. above the sample median). The annotation box reports the regression coefficients for PGS, IL-10 and their interaction term, as well as the proportion of CMI variance explained by the model (R^2^).

Lastly, Figure 5 shows the results of the global regression model evaluating the interaction between the Pro-inflammatory Gradient Score (PGS) and IL-10 in predicting CMI in the total sample of older adults. In this analysis, both PGS and IL-10 were included as z-standardized continuous variables together with their interaction term (z-PGS × z-IL-10). Across the full range of the pro-inflammatory axis, higher PGS values were associated with higher predicted CMI; however, the slope of this relationship differed according to IL-10 levels. Among individuals with low IL-10, CMI increased more steeply along the PGS gradient, whereas among those with high IL-10 the rise in CMI was attenuated, resulting in a visibly flatter slope for the same inflammatory burden. Consistent with this graphical pattern, the PGS × IL-10 interaction term was negative and statistically significant (p < 0.001), indicating that higher IL-10 weakens the association between pro-inflammatory activation and cardiometabolic load. Overall, the model explained approximately 48% of the variance in CMI (R^2^ = 0.48), supporting a buffering role of IL-10 in the CMI–inflammation relationship.

## 4 Discussion

In the present study, the cohort of older adults participating showed that higher values of the Cardiometabolic Index (CMI) were consistently associated with a more adverse cardiometabolic profile, since the volunteers in the higher CMI group presented higher BMI, a poor lipid profile (total cholesterol, LDL-c, triglycerides and TG/HDL-c ratio), and a higher prevalence of hypertension and cardiovascular disease compared with those in the lower CMI group, in both sexes (Luo et al., 2025). Thus, this pattern supports the view that CMI is able to capture the convergence of central adiposity and dyslipidemia, then putatively serving as an integrating index of cardiometabolic stress that may be more sensitive than BMI alone in the context of aging (Luo et al., 2025). At the same time, between-group differences in absolute cytokine concentrations were relatively modest, which is consistent with the concept of inflammaging (Franceschi et al., 2018), since in older individuals, inflammation usually appears as a chronic, diffuse, low-grade elevation with multiple mediators, rather than as pronounced peaks of any single cytokine. Under these conditions, the overall architecture of the cytokine network and its interaction with cardiometabolic load may be more informative than isolated measurements.

It is worth citing that the multiple linear regression analyses, stratified by sex, nutritional status, and CMI category, add important data of mechanistic information to this broader picture. In this respect, in the eutrophic older men subgroup, IL-6 and IL-10 consistently remained in the final models for both lower and higher CMI strata, which, in conjunction, can explain a relevant proportion of CMI variance. In fact, this pattern can suggest that, in older men with normal weight, CMI is involved in a cytokine milieu characterized by the interplay between IL-6 and IL-10 rather than dominated by isolated “spikes” of individual markers.

Regarding IL-6, the literature highlights that it is a pleiotropic cytokine with both pro-inflammatory and regulatory functions, depending on its concentration, cellular source, and signaling pathway (classic versus trans-signaling) (Xu et al., 2017). Specifically in cardiometabolic settings, chronically elevated IL-6 has been associated with central adiposity, insulin resistance, endothelial dysfunction, and increased cardiovascular risk, reflecting sustained activation of adipose and vascular compartments (Tylutka et al., 2024). Beyond these features, IL-6 also participates in metabolic adaptation to physical exercise and in acute-phase responses that can have short-term protective roles. Therefore, the simultaneous presence of IL-6 and IL-10, particularly found in the older men with normal weight subgroup, may suggest that CMI is influenced not by an uncontrolled inflammatory state, but by a dynamic balance in which IL-6–mediated inflammatory signals are at least partially counter-regulated by IL-10 (Li et al., 2023).

Interestingly, in the older men subgroup with obesity and high CMI, the pattern shifts towards a more clearly atherogenic, Th1-oriented profile, with TNF-α and IFN-γ emerging as the main predictors of CMI. In agreement with the literature, TNF-α is a central mediator of obesity-related metaflammation, since it promotes insulin resistance in the liver and skeletal muscle, impairs insulin signaling in adipocytes, increases lipolysis and free fatty acid flux to the liver, and contributes to endothelial activation and vascular dysfunction (Wu and Ballantyne, 2020). In terms of IFN-γ, it is a prototypical Th1 cytokine that enhances macrophage activation, upregulates antigen presentation, and supports a more pro-atherogenic, plaque-activating environment in the arterial wall (Ajoolabady et al., 2024). Hence, the co-occurrence of TNF-α and IFN-γ as significant predictors in older men with obesity and high CMI is therefore biologically plausible and consistent with a state of advanced cardiometabolic remodeling (Li et al., 2025), in which adipose tissue, immune cells, and the vascular site form a tightly coupled inflammatory circuit. In this context, CMI appears to reflect not only abdominal adiposity and dyslipidemia, but also the intensity of a Th1-skewed inflammatory response linked to cardiometabolic damage.

Regarding the older women group, the findings showed that even though the backward-selected models were more modest and less stable associations, the overall pattern is still informative. Of note, in the older women subgroup with normal weight and lower CMI, IL-1β and TNF-α tended to remain in the final models, with borderline p-values, which can suggest that, even in the absence of obesity, a subtle activation of inflammasome-related and also TNF-α–mediated pathways can already be present in cardiometabolic burden conditions. Particularly, IL-1β is tightly linked to NLRP3 inflammasome activation in adipose tissue, endothelium, and vascular macrophages, and sustained IL-1β production has been implicated in atherosclerosis, type 2 diabetes, and functional decline in older adults (Jiao and Tian, 2025). Similarly, TNF-α contributes to endothelial dysfunction, increased vascular stiffness, and impaired insulin signaling (Baaten et al., 2023; Herzog et al., 2025). Besides these evidences, whereas the older women group with obesity showed that tha regression models were weaker, the broader set of findings in this study, respresented by higher mean levels of IL-1β, IL-6, IFN-γ and TNF-α, increased IL-1β/IL-10 and IFN-γ/IL-10 ratios, and stronger correlations between CMI and pro-inflammatory markers, consistently points to a cardiometabolic environment in which excess adiposity may amplify both inflammasome activation and Th1-type responses. Taken together, these results suggest that, in older women, the association between CMI and cytokines may be expressed less through a single “dominant” cytokine in the models and more through a global shift of the network towards a chronic low-grade inflammatory state, with prominent reliance on regulatory mechanisms to prevent cumulative tissue damage (Arakelyan et al., 2025).

In order to improve the comprehension of these networks, we constructed a Pro-inflammatory Gradient Score (PGS) incorporating IL-1β, IL-6, TNF-α, and IFN-γ. This approach condenses redundant information into a single axis of “inflammatory pressure” (Malenica et al., 2023). Similar composite inflammatory scores, derived from PCA or from aggregated protein panels, have been associated with cardiometabolic risk factors and with outcomes such as incident hypertension, diabetes, cardiovascular disease, and mortality in large cohorts (Tejada et al., 2022). Here, PGS can be interpreted as a continuous gradient of inflammatory activation integrating these well-known pro-inflammatory cytokines, all closely associated with insulin resistance, endothelial dysfunction, extracellular matrix remodeling, and lipid metabolism alterations. The stronger association between PGS and CMI in the volunteer subgroups with obesity can suggest that comparable levels of inflammatory activation may have more adverse cardiometabolic consequences when superimposed on the mechanical and metabolic overload imposed by central adiposity (Fong et al., 2024).

Based on this inflammatory “architecture”, the cytokines with anti-inflammatory properties, such as IL-10, play a pivotal role as a modulatory and “buffering” mediator. In terms of IL-10, it is produced by multiple immune cell populations and is closely related to the polarization of macrophages towards less inflammatory phenotypes, the inhibition of IL-6, TNF-α, and IFN-γ production, and protection against chronic tissue injury in models of obesity, insulin resistance, and cardiovascular disease. Studies in human adipose tissue have reported associations between IL-10 expression or secretion and metabolic parameters, including insulin sensitivity, suggesting that, under metabolic stress, the ability of adipose tissue to mount an IL-10 response may influence the transition from balanced to unbalanced inflammation (Acosta et al., 2019).

Our findings align with this suggestion by indicating that IL-10 does not supposedly act by controlling pro-inflammatory cytokine expressions, but more importantly by modulating how a given inflammatory burden is converted into lower or higher CMI (Xu et al., 2021) through dynamically controlling the intensity and duration of inflammatory responses (Carlini et al., 2023). Here, when CMI was modulated as a function of PGS and IL-10 as continuous variables, a consistent pattern emerged in volunteer groups (both older men and older women), since for a similar level of PGS, individuals with higher IL-10 showed a shallower slope in the relationship between inflammation and CMI, whereas those with lower IL-10 exhibited a steeper CMI increase across the same inflammatory gradient. Based on these pieces of evidence, it is plausible to suppose that IL-10 acted as a “buffer” that attenuated the impact of inflammatory pressure on cardiometabolic status.

These findings are consistent with recent evidence demonstrating that IL-10 exerts dose-dependent protective effects by suppressing the expression of key pro-inflammatory cytokines (TNF-α, IL-1β, IL-6) and reducing macrophage-derived reactive oxygen species production, thereby promoting a shift from inflammatory to anti-inflammatory macrophage phenotypes (Zhang et al., 2025), while simultaneously stabilizing atherosclerotic plaques through reduced recruitment and activation of pro-inflammatory immune cells (Sukhorukov et al., 2025). Taken together, these mechanisms can reinforce the suggestion that IL-10 is a corollary agent involved in the control/regulation of inflammation, thus dampening the translation of inflammatory burden into cardiometabolic risk (Saxton et al., 2021).

Interestingly, in the older women group, this IL-10 regulatory effect was sufficiently strong for this cytokine to appear as an independent predictor in some of the multivariable models, whereas in the older men group the modulatory role of IL-10 was more evident through its interaction with the pro-inflammatory component (PGS), even when IL-10 did not remain as an isolated predictor after backward selection. Therefore, these sex-dependent patterns are consistent with evidence that inflammatory markers, including IL-10, show stronger associations with adverse health outcomes in older women compared to older men (Yang et al., 2025), and align with reports that older women exhibit greater capacity for immune counter-regulation and IL-10-mediated responses than older men (Calabrò et al., 2023).

It is of utmost importance to point out that this sex-dependent pattern agrees with evidence in which there is sexually dimorphic immune and metabolic responses, with distinct age-related trajectories of immune cell composition and inflammatory activation (Gheitasi et al., 2025), where women often display stronger immune activation but also a greater dependence on regulatory pathways to limit chronic tissue damage due to enhanced inflammatory trajectories with aging (Milan-Mattos et al., 2019; Müller et al., 2025). In fact, our results allow us to suggest that, in the older men group, the coupling between pro-inflammatory cytokines and CMI is more direct, so that reductions in inflammatory burden and central adiposity may translate more straightforwardly into improved cardiometabolic risk. By contrast, in the older women group, sexual dimorphism in cardiometabolic disease pathogenesis (Chevalley et al., 2025) means that a similar magnitude of inflammatory activation may lead to different CMI trajectories depending on the regulatory capacity associated with IL-10, thus suggesting that two women with comparable inflammatory gradients may exhibit quite distinct cardiometabolic profiles if they differ in the magnitude of their anti-inflammatory response.

In a clinical context, our findings not only reinforce the potential of CMI as a simple, integrative index calculated from readily available anthropometric and laboratory data, validated in machine learning models (Luo et al., 2025) to identify the cardiometabolic risk, but also can potentially indicate that CMI may be an interesting tool that allow to better understanding conditions in which metabolic and inflammatory stress converging to a higher-risk profile. Incorporating inflammatory information, whether through composite axes such as PGS or through simple patterns combining inflammatory mediators, both with pro- and anti-inflammatory properties, may further refine risk stratification in the same manner that composite biomarker indices provide superior predictive value to individual markers (Zhou et al., 2025) by identifying subgroups in which a buffering capacity is compromised and who might therefore benefit from more intensive interventions. Evidence from randomized controlled trials demonstrates that comprehensive lifestyle modifications targeting weight control, diet quality, physical activity, and optimization of comorbidities effectively reduce cardiometabolic risk factors and biological aging acceleration in older adults (Cai et al., 2019; Liu et al., 2021), with multi-dimensional assessment approaches providing superior identification of at-risk individuals for early intervention (Zeng et al., 2025).

**FIGURE 6:**
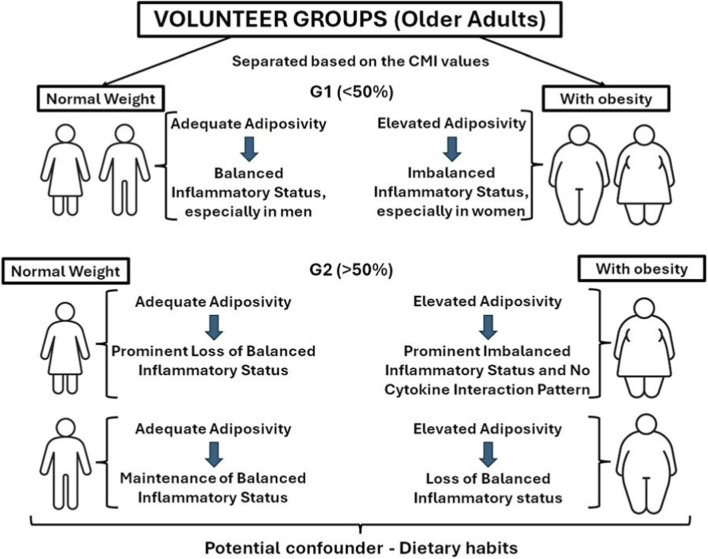
Schematic representation of the main findings of this study.

In the present study, we were able to demonstrate that even though the isolated cytokine assessment presents valuable data, the integrative analysis performed here offers a plausible and testable mechanistic framework for understanding how inflammaging, obesity, and cardiometabolic risk interact in older men and women, extending previous pilot observations focused on older men (Carvalho et al., 2024). In this framework, CMI emerges not only as a metabolic index but as an accessible index of the balance between the intensity of inflammatory signaling and the effectiveness of IL-10-mediated counter-regulation. Further longitudinal studies with larger samples, more detailed immune phenotyping, and better descriptions of clinical endpoints will be crucial to determine whether the buffering role of IL-10 in the associations between inflammation and CMI translates into tangible differences in cardiometabolic events and functional decline over time. Such studies may also clarify whether combining CMI with inflammatory profiles can ultimately be incorporated into risk stratification strategies and personalized interventions in older adults. The main findings are summarized in Figure 6.

Lastly, several limitations should be mentioned, such as: (World Health Organization, 2025a) the cross-sectional design, which precludes causal inference regarding the associations among CMI, inflammation, and cardiometabolic outcomes, as temporal precedence of causes and effects cannot be established (Taris et al., 2021; Samir and Levin, 2013) the sample size is relatively modest, particularly when stratifying simultaneously by sex, nutritional status and CMI categories, which reduces the stability of some models and increases the possibility of unstable or chance findings in smaller subgroups, a recognized limitation when conducting multiple simultaneous stratifications (Wang et al., 2024; Lee et al., 2014) although the cytokine panel is relatively broad, it cannot fully represent the complexity of immune responses in aging, since involves multiple immunological alterations, including epigenetic dysregulation, thymic involution, T cell receptor diversity skewing, and dynamic shifts in immune cell compositions occur simultaneously and interact in ways that single-timepoint systemic cytokine measurements cannot fully represent the inflammaging (Upadhyay et al., 2025); (Stoodley and Conroy, 2024) the lack of evaluations of other mediators, such as hormonal and tissue-level markers, which may play important roles in the aging context; and (Culberson et al., 2023) the cohort derives from a specific context and may not be fully representative of other populations with different ethnic, socioeconomic, or clinical profiles.

## 5 Conclusion

In summary, this study supports the Cardiometabolic Index as a simple, accessible tool that integrates not only central adiposity, dyslipidemia, and cardiometabolic risk, but also the low-grade inflammation in older adults, while revealing a mechanistic framework in which CMI could also reflect the systemic inflammatory status, particularly that involved in the (un)balance between pro-inflammatory cytokine and IL-10–mediated counter-regulation. Additionally, sex-dependent patterns further indicated more direct inflammation-CMI coupling in older men and greater reliance on IL-10–driven regulatory capacity in older women.

Cardiometabolic index (CMI) was calculated by multiplying the triglycerides/HDL-cholesterol (TG/HDL-C) ratio by the waist/height ratio, as shown in Equation 1 that follows:

$$CMI=\left(\frac{TG}{HDL-c}\right)\;X\;\left(\frac{WC}{Height}\right)$$ (1)

Note: TG = triglycerides in mg/dL; HDL-c = high-density lipoprotein cholesterol in mg/dL; Wc = waist circumference in centimeters; and height in meters.

## Data Availability

The original contributions presented in the study are included in the article/Supplementary Material, further inquiries can be directed to the corresponding author.

## Supplementary material

SUPPLEMENTARY FIGURE S1 Discriminative performance of the Cardiometabolic Index (CMI) for obesity and high pro-inflammatory burden, stratified by sex. **(A,B)** show ROC curves for continuous CMI as a predictor of obesity (yes/no, defined by body composition criteria) in older men **(A)** and older women **(B)**, respectively. **(C,D)** show ROC curves for continuous CMI as a predictor of a high pro-inflammatory gradient score (PGS) in older men **(C)** and older women **(D)**. In all panels, the solid line represents the ROC curve generated from all possible CMI cut-offs, and the diagonal dashed line represents the line of no discrimination (AUC = 0.5). The x-axis shows 1 − specificity and the y-axis sensitivity. The inset in each panel reports the corresponding area under the curve (AUC) and p-value (all p < 0.001).
