# Supplementary figures and images for: New insights into the association between cardiometabolic index with metabolic profile, nutritional status, and inflammaging in older adults
Source: Front Aging. 2026 Jan 12;6:1699767. doi: 10.3389/fragi.2025.1699767 (PMC12833520; doi:10.3389/fragi.2025.1699767)

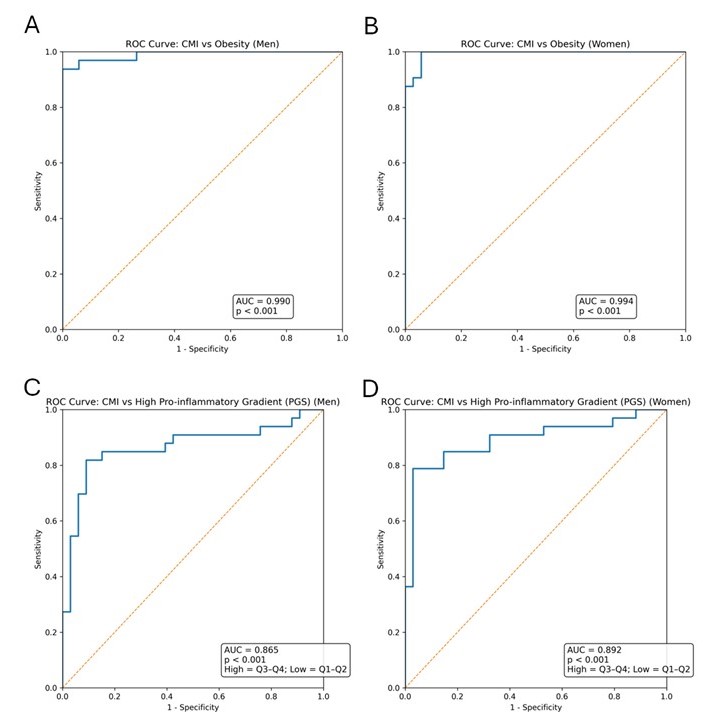

Supplement: Supplementary file 1 [file Image1.jpeg]
