# Supplementary material for: New insights into the association between cardiometabolic index with metabolic profile, nutritional status, and inflammaging in older adults
Source: Front Aging. 2026 Jan 12;6:1699767. doi: 10.3389/fragi.2025.1699767 (PMC12833520; doi:10.3389/fragi.2025.1699767)
Supplement: Supplementary file 2 [file DataSheet1.pdf]

Supplementary Data

Contents

- Supplementary Methods
- Abbreviations and Grouping Schema
- Section A — Simple Linear Regressions: CMI vs. Physical Activity and CMI vs. Smoking
- Section B — Multiple Linear Regression: Physical Activity + Smoking → CMI
- Section C — Cytokine Models: Physical Activity + Smoking → Cytokines
- Section D — Backward Stepwise Regression: Cytokines → CMI (tables)

Supplementary Methods

Analyses were performed in Python (3.11) using Jupyter Notebooks. Data wrangling used pandas (2.1.4) and numpy (1.26.2). Linear models were fit by ordinary least squares via statsmodels (0.14.1). For backward stepwise selection, the least-informative predictor was iteratively removed until only variables with $p < 0.15$ remained. We report β, 95% confidence intervals (CI), p-values, R^2/adjusted R^2, and final-model F and p(F). Figures (when applicable) were produced with matplotlib (3.8.2) and seaborn (0.13.0). Word reports were prepared with python-docx (0.8.11).

Abbreviations and Grouping Schema

- CMI: Cardiometabolic Index
- G1/G2: Lower/higher CMI subgroups within each sex × nutritional-status stratum
- OM: Older Men; OW: Older Women
- β: Standardized regression coefficient; CI: Confidence Interval; SE: Standard Error
- Predictors: Physical Activity and Smoking (no shorthand used)

Section A — Simple Linear Regressions

Outcome: CMI. Predictor: Physical Activity or Smoking. Design: Older Men/Older Women × Normal weight/Obesity; overall and G1/G2 strata.

- **Older Men — Normal weight (All, N=34)**

• Physical Activity → CMI: β=0.167, 95% CI [−0.156; 0.489], R^2=0.033 (adj 0.003), F(1,32)=1.107, p=0.3006.

• Smoking → CMI: β=−0.151, 95% CI [−0.523; 0.221], R^2=0.021 (adj −0.010), F(1,32)=0.683, p=0.4148.

- **Older Men — Normal weight (G1, N=17)**

• Physical Activity → CMI: β=−0.147, 95% CI [−0.368; 0.074], R^2=0.180 (adj 0.098), F=2.191, p=0.1696.

• Smoking → CMI: β=−0.098, 95% CI [−0.333; 0.136], R^2=0.081 (adj −0.011), F=0.877, p=0.3711.

- **Older Men — Normal weight (G2, N=17)**

• Physical Activity → CMI: β=−0.057, 95% CI [−0.336; 0.222], R^2=0.012 (adj −0.050), F=0.187, p=0.6710.

• Smoking → CMI: β=0.075, 95% CI [−0.300; 0.449], R^2=0.011 (adj −0.051), F=0.179, p=0.6783.

- **Older Men — Obesity (All, N=30)**

• Physical Activity → CMI: β=0.016, 95% CI [−0.026; 0.059], R^2=0.021 (adj −0.013), F=0.613, p=0.4400.

• Smoking → CMI: β=0.010, 95% CI [−0.097; 0.116], R^2=0.001 (adj −0.033), F=0.035, p=0.8538.

- **Older Men — Obesity (G1, N=15)**

• Physical Activity → CMI: β=−0.003, 95% CI [−0.074; 0.067], R^2=0.001 (adj −0.076), F=0.009, p=0.9265.

• Smoking → CMI: Predictor without variance (dropped).

- **Older Men — Obesity (G2, N=15)**

• Physical Activity → CMI: β=0.046, 95% CI [−0.014; 0.106], R^2=0.176 (adj 0.113), F=2.780, p=0.1194.

• Smoking → CMI: β=−0.004, 95% CI [−0.122; 0.113], R^2≈0.000 (adj −0.076), F=0.006, p=0.9381.

- **Older Women — Normal weight (All, N=34)**

• Physical Activity → CMI: β=−0.314, 95% CI [−0.731; 0.103], R^2=0.067 (adj 0.038), F=2.351, p=0.1347.

• Smoking → CMI: β=0.252, 95% CI [−0.188; 0.692], R^2=0.040 (adj 0.010), F=1.357, p=0.2524.

- **Older Women — Normal weight (G1, N=17)**

• Physical Activity → CMI: β=−0.260, 95% CI [−0.707; 0.187], R^2=0.081 (adj 0.027), F=1.506, p=0.2365.

• Smoking → CMI: β=0.481, 95% CI [−0.013; 0.975], R^2=0.199 (adj 0.152), F=4.217, p=0.0557.

- **Older Women — Normal weight (G2, N=17)**

• Physical Activity → CMI: β=−0.014, 95% CI [−0.942; 0.913], R^2≈0.000 (adj −0.071), F≈0.001, p=0.9744.

• Smoking → CMI: β=−0.038, 95% CI [−0.746; 0.670], R^2≈0.001 (adj −0.070), F=0.013, p=0.9104.

- **Older Women — Obesity (All, N=34)**

• Physical Activity → CMI: β=0.037, 95% CI [−0.512; 0.585], R^2≈0.001 (adj −0.033), F=0.019, p=0.8923.

• Smoking → CMI: Predictor without variance (dropped).

- **Older Women — Obesity (G1, N=17)**

• Physical Activity → CMI: β=0.176, 95% CI [−0.507; 0.859], R^2=0.020 (adj −0.046), F=0.302, p=0.5905.

• Smoking → CMI: Predictor without variance (dropped).

- **Older Women — Obesity (G2, N=17)**

• Physical Activity → CMI: β=−0.149, 95% CI [−1.114; 0.816], R^2=0.009 (adj −0.068), F=0.111, p=0.7438.

• Smoking → CMI: Predictor without variance (dropped).

Section B — Multiple Linear Regression: Physical Activity + Smoking → CMI

Outcome: CMI. Predictors: Physical Activity and Smoking. Design: Men/Women × Eutrophic/Obese; overall and G1/G2.

- **Older Men — Normal weight (All, N=34)**

• R^2=0.047 (adj −0.014), F=0.772, p(F)=0.4710.

• Physical Activity: β=0.150 (0.162), 95% CI [−0.180; 0.480], p=0.3599.

• Smoking: β=−0.125 (0.185), 95% CI [−0.503; 0.253], p=0.5050.

- **Older Men — Normal weight (G1, N=17)**

• R^2=0.295 (adj 0.138), F=1.884, p(F)=0.2073.

• Physical Activity: β=−0.162 (0.098), 95% CI [−0.383; 0.059], p=0.1324.

• Smoking: β=−0.119 (0.098), 95% CI [−0.340; 0.103], p=0.2558.

- **Older Men — Normal weight (G2, N=17)**

• R^2=0.020 (adj −0.111), F=0.150, p(F)=0.8616.

• Physical Activity: β=−0.050 (0.137), 95% CI [−0.341; 0.242], p=0.7213.

• Smoking: β=0.065 (0.184), 95% CI [−0.327; 0.456], p=0.7296.

- **Older Men — Obesity (All, N=30)**

• R^2=0.023 (adj −0.047), F=0.333, p(F)=0.7196.

• Physical Activity: β=0.017 (0.021), 95% CI [−0.027; 0.061], p=0.4333.

• Smoking: β=0.014 (0.053), 95% CI [−0.094; 0.122], p=0.7897.

- **Older Men — Obesity (G1, N=15)**

• R^2=0.001 (adj −0.076), F=0.009, p(F)=0.9265.

• Physical Activity: β=−0.003 (0.033), 95% CI [−0.074; 0.067], p=0.9265.

• Smoking: Predictor without variance (dropped).

- **Older Men — Obesity (G2, N=15)**

• R^2=0.176 (adj 0.113), F=2.780, p(F)=0.1194.

• Physical Activity: β=0.046 (0.028), 95% CI [−0.014; 0.106], p=0.1194.

• Smoking: β=−0.004 (0.053), 95% CI [−0.122; 0.113], p=0.9381.

- **Older Women — Normal weight (All, N=34)**

• R^2=0.067 (adj 0.038), F=2.351, p(F)=0.1347.

• Physical Activity: β=−0.314 (0.205), 95% CI [−0.731; 0.103], p=0.1347.

• Smoking: β=0.252 (0.216), 95% CI [−0.188; 0.692], p=0.2524.

- **Older Women — Normal weight (G1, N=17)**

• R^2=0.199 (adj 0.089), F=2.109, p(F)=0.1499.

• Physical Activity: β=−0.260 (0.213), 95% CI [−0.707; 0.187], p=0.2365.

• Smoking: β=0.481 (0.234), 95% CI [−0.013; 0.975], p=0.0557.

- **Older Women — Normal weight (G2, N=17)**

• R^2≈0.000 (adj −0.141), F=0.006, p(F)=0.9936.

• Physical Activity: β=−0.015 (0.342), 95% CI [−0.757; 0.727], p=0.9668.

• Smoking: β=−0.018 (0.304), 95% CI [−0.671; 0.635], p=0.9544.

- **Older Women — Obese (All, N=34)**

• R^2=0.001 (adj −0.033), F=0.019, p(F)=0.8923.

• Physical Activity only (Smoking no variance): β=0.037 (0.269), 95% CI [−0.512; 0.585], p=0.8923.

- **Older Women — Obesity (G1, N=17)**

• R^2=0.020 (adj −0.046), F=0.302, p(F)=0.5905.

• Physical Activity only (Smoking no variance): β=0.176 (0.320), 95% CI [−0.507; 0.859], p=0.5905.

- **Older Women — Obesity (G2, N=17)**

• R^2=0.009 (adj −0.068), F=0.111, p(F)=0.7438.

• Physical Activity only (Smoking no variance): β=−0.149 (0.446), 95% CI [−1.114; 0.816], p=0.7438.

Section C — Cytokine Models: Physical Activity + Smoking → Cytokines

Outcome: each cytokine (IL-1β, IL-6, IL-10, TNF-α, IFN-γ). Predictors: Physical Activity and Smoking. Design: Men/Women × Eutrophic/Obese; overall models per stratum.

- **Older Men — Normal weight (N=34)**

• IL-1β: R^2=0.055 (adj −0.040), F=0.579, p(F)=0.6336. Physical Activity β=0.141 (p=0.3986); Smoking β=−0.134 (p=0.4819).

• IL-6: R^2=0.118 (adj 0.029), F=1.333, p(F)=0.2821. Physical Activity β=0.085 (p=0.6072); Smoking β=−0.175 (p=0.3497).

• IL-10: R^2=0.062 (adj −0.031), F=0.665, p(F)=0.5803. Physical Activity β=0.127 (p=0.4514); Smoking β=−0.146 (p=0.4471).

• TNF-α: R^2=0.062 (adj −0.032), F=0.656, p(F)=0.5855. Physical Activity β=0.137 (p=0.4100); Smoking β=−0.140 (p=0.4618).

• IFN-γ: R^2=0.056 (adj −0.038), F=0.595, p(F)=0.6230. Physical Activity β=0.141 (p=0.3972); Smoking β=−0.128 (p=0.4991).

- **Older Men — Obesity (N=30)**

• IL-1β: R^2=0.078 (adj −0.029), F=0.729, p(F)=0.5442. Physical Activity β=0.028 (p=0.2211); Smoking β=0.010 (p=0.8511).

• IL-6: R^2=0.057 (adj −0.051), F=0.527, p(F)=0.6674. Physical Activity β=0.027 (p=0.2363); Smoking β=0.013 (p=0.8046).

• IL-10: R^2=0.109 (adj 0.006), F=1.056, p(F)=0.3847. Physical Activity β=0.019 (p=0.4067); Smoking β=0.016 (p=0.7545).

• TNF-α: R^2=0.242 (adj 0.155), F=2.771, p(F)=0.0616. Physical Activity β=0.028 (p=0.1677); Smoking β=0.009 (p=0.8492).

• IFN-γ: R^2=0.088 (adj −0.017), F=0.840, p(F)=0.4843. Physical Activity β=0.031 (p=0.1768); Smoking β=0.014 (p=0.7945).

- **Older Women — Normal weight (N=34)**

• IL-1β: R^2=0.141 (adj 0.058), F=1.693, p(F)=0.1889. Physical Activity β=35.548 (p=0.4897); Smoking β=65.821 (p=0.2276).

• IL-6: R^2=0.067 (adj −0.024), F=0.739, p(F)=0.5366. Physical Activity β=23.811 (p=0.6546); Smoking β=67.442 (p=0.2503).

• IL-10: R^2=0.095 (adj 0.007), F=1.081, p(F)=0.3716. Physical Activity β=27.936 (p=0.5946); Smoking β=58.610 (p=0.2913).

• TNF-α: R^2=0.064 (adj −0.026), F=0.712, p(F)=0.5525. Physical Activity β=42.298 (p=0.4466); Smoking β=50.188 (p=0.3687).

• IFN-γ: R^2=0.067 (adj −0.023), F=0.741, p(F)=0.5358. Physical Activity β=34.743 (p=0.5198); Smoking β=43.773 (p=0.4313).

- **Older Women — Obesity (N=34)**

• IL-1β: R^2=0.033 (adj −0.033), F=0.500, p(F)=0.6114. Physical Activity β=74.170 (p=0.4850); Smoking: dropped (no variance).

• IL-6: R^2=0.022 (adj −0.046), F=0.319, p(F)=0.7291. Physical Activity β=74.449 (p=0.4862); Smoking: dropped.

• IL-10: R^2=0.016 (adj −0.052), F=0.241, p(F)=0.7876. Physical Activity β=71.844 (p=0.5059); Smoking: dropped.

• TNF-α: R^2=0.032 (adj −0.035), F=0.479, p(F)=0.6242. Physical Activity β=76.356 (p=0.4731); Smoking: dropped.

• IFN-γ: R^2=0.017 (adj −0.051), F=0.243, p(F)=0.7856. Physical Activity β=73.340 (p=0.4938); Smoking: dropped.

Section D — Backward Stepwise Regression: Cytokines → CMI

Values in this section are derived directly from the original backward-stepwise report. For each subgroup, we present: (i) the initial multivariable model; (ii) stepwise elimination with β from the initial model for removed cytokines; and (iii) the final model with fit metrics.

- **Older Men — Normal weight (G1: <50% CMI)**

Initial multivariable model (R^2 = 0.365):

Cytokine	B	95% CI (lower)	95% CI (upper)	p-value
IL-1β	0.0353	-0.2777	0.3483	0.8085

IL-6	-0.1342	-0.2585	-0.01	0.0366
IL-10	0.0767	-0.0705	0.224	0.2758
IFN-γ	-0.0255	-0.1062	0.0553	0.5018
TNF-α	0.0176	-0.0873	0.1224	0.7192

Backward elimination steps (β shown from initial model):

**Step**	**Removed cytokine**	**β (initial)**	**95% CI (lower)**	**95% CI (upper)**	**p at removal**	**R^2 after removal**
1	IL-1β	0.0353	-0.2777	0.3483	0.8085	0.362
2	IFN-γ	-0.0255	-0.1062	0.0553	0.5043	0.337
3	TNF-α	0.0176	-0.0873	0.1224	0.6418	0.325

Final model:

**Cytokine**	**B**	**p-value**
IL-6	-0.1333	**0.0215**
IL-10	0.0836	**0.0245**

Final fit: R^2 = 0.325; F = 3.369; p(F) = 0.063893

- **Older Men — Normal weight (G2: >50% CMI)**

Initial multivariable model (R^2 = 0.551):

**Cytokine**	**B**	**95% CI (lower)**	**95% CI (upper)**	**p-value**
IL-1β	-0.0078	-0.1293	0.1137	0.8901
IL-6	-0.3691	-0.5948	-0.1435	**0.0042**
IL-10	0.128	-0.0073	0.2633	0.0615
IFN-γ	0.0109	-0.0535	0.0754	0.7157
TNF-α	0.0443	-0.1139	0.2025	0.55

Backward elimination steps (β shown from initial model):

Step	Removed cytokine	β (initial)	95% CI (lower)	95% CI (upper)	p at removal	R^2 after removal
1	IL-1β	-0.0078	-0.1293	0.1137	0.8901	0.55
2	IFN-γ	0.0109	-0.0535	0.0754	0.7038	0.544
3	TNF-α	0.0443	-0.1139	0.2025	0.4626	0.524

Final model:

Cytokine	B	p-value
IL-6	-0.3601	**0.0016**
IL-10	0.1547	**0.0019**

Final fit: R^2 = 0.524; F = 7.711; p(F) = 0.005522

- **Older Men — Obesity (G1: <50% CMI)**

Initial multivariable model (R^2 = 0.164):

Cytokine	B	95% CI (lower)	95% CI (upper)	p-value
IL-1β	-0.0182	-0.3481	0.3118	0.9035
IL-6	0.0996	-0.2129	0.4121	0.4891
IL-10	-0.0236	-0.5379	0.4906	0.9195
IFN-γ	0.0524	-0.1921	0.297	0.6393
TNF-α	-0.0836	-0.4222	0.2549	0.59

Backward elimination steps (β shown from initial model):

Step	Removed cytokine	β (initial)	95% CI (lower)	95% CI (upper)	p at removal	R^2 after removal
1	IL-10	-0.0236	-0.5379	0.4906	0.9195	0.163
2	IL-1β	-0.0182	-0.3481	0.3118	0.7785	0.156
3	IFN-γ	0.0524	-0.1921	0.297	0.4592	0.111

4	TNF-α	-0.0836	-0.4222	0.2549	0.6243	0.092

Final model:

**Cytokine**	**B**	**p-value**
IL-6	0.0879	0.2722

Final fit: R^2 = 0.092; F = 1.315; p(F) = 0.272216

- **Older Men — Obesity (G2: >50% CMI)**

Initial multivariable model (R^2 = 0.643):

**Cytokine**	**B**	**95% CI (lower)**	**95% CI (upper)**	**p-value**
IL-1β	-0.1468	-0.4161	0.1224	0.2486
IL-6	0.2217	-0.1856	0.629	0.2495
IL-10	0.0108	-0.0871	0.1087	0.808
IFN-γ	0.2739	0.0503	0.4975	**0.0217**
TNF-α	0.1381	0.0419	0.2342	**0.01**

Backward elimination steps (β shown from initial model):

**Step**	**Removed cytokine**	**β (initial)**	**95% CI (lower)**	**95% CI (upper)**	**p at removal**	**R^2 after removal**
1	IL-10	0.0108	-0.0871	0.1087	0.808	0.64
2	IL-6	0.2217	-0.1856	0.629	0.2329	0.582
3	IL-1β	-0.1468	-0.4161	0.1224	0.4151	0.555

Final model:

**Cytokine**	**β**	**p-value**
IFN-γ	0.207	**0.026**
TNF-α	0.1309	**0.0072**

Final fit: R^2 = 0.555; F = 7.489; p(F) = 0.007744

- **Older Women — Normal weight (G1: <50% CMI)**

Initial multivariable model (R^2 = 0.354):

Cytokine	B	95% CI (lower)	95% CI (upper)	p-value
IL-1β	-0.2162	-0.455	0.0226	0.072
IL-6	0.0609	-0.3002	0.4221	0.7195
IL-10	-0.0692	-0.2838	0.1454	0.4957
IFN-γ	0.0491	-0.0165	0.1147	0.1288
TNF-α	0.1093	-0.037	0.2555	0.1296

Backward elimination steps (β shown from initial model):

Step	Removed cytokine	β (initial)	95% CI (lower)	95% CI (upper)	p at removal	R^2 after removal
1	IL-6	0.0609	-0.3002	0.4221	0.7195	0.346
2	IL-10	-0.0692	-0.2838	0.1454	0.5454	0.327

Final model:

Cytokine	B	p-value
IL-1β	-0.2453	**0.0214**
IFN-γ	0.0447	0.129
TNF-α	0.1065	0.083

Final fit: R^2 = 0.327; F = 2.268; p(F) = 0.125408

- **Older Women — Normal weight (G2: >50% CMI)**

Initial multivariable model (R^2 = 0.071):

Cytokine	B	95% CI (lower)	95% CI (upper)	p-value
IL-1β	-0.0411	-0.2264	0.1441	0.6348
IL-6	0.0073	-0.2144	0.2291	0.9432

IL-10	0.0046	-0.1552	0.1644	0.9502
IFN-γ	0.0844	-0.2042	0.373	0.533
TNF-α	-0.0157	-0.2018	0.1703	0.8557

Backward elimination steps (β shown from initial model):

Step	Removed cytokine	β (initial)	95% CI (lower)	95% CI (upper)	p at removal	R^2 after removal
1	IL-10	0.0046	-0.1552	0.1644	0.9502	0.07
2	IL-6	0.0073	-0.2144	0.2291	0.8877	0.069
3	TNF-α	-0.0157	-0.2018	0.1703	0.8342	0.065
4	IL-1β	-0.0411	-0.2264	0.1441	0.5932	0.046

Final model:

Cytokine	B	p-value
IFN-γ	0.0467	0.4109

Final fit: R^2 = 0.046; F = 0.716; p(F) = 0.410881

- **Older Women — Obesity (G1: <50% CMI)**

Initial multivariable model (R^2 = 0.456):

Cytokine	B	95% CI (lower)	95% CI (upper)	p-value
IL-1β	0.0389	-0.08	0.1577	0.4829
IL-6	-0.0175	-0.1979	0.1628	0.8332
IL-10	-0.014	-0.1132	0.0852	0.7591
IFN-γ	0.0816	-0.0433	0.2065	0.1763
TNF-α	0.0015	-0.181	0.184	0.9859

Backward elimination steps (β shown from initial model):

Step	Removed cytokine	β (initial)	95% CI (lower)	95% CI (upper)	p at removal	R^2 after removal

1	TNF-α	0.0015	-0.181	0.184	0.9859	0.456
2	IL-6	-0.0175	-0.1979	0.1628	0.821	0.454
3	IL-10	-0.014	-0.1132	0.0852	0.4752	0.429
4	IL-1β	0.0389	-0.08	0.1577	0.3959	0.395

Final model:

**Cytokine**	**B**	**p-value**
IFN-γ	0.0897	**0.0091**

Final fit: $R^2 = 0.395$; F = 9.144; p(F) = 0.00911

- **Older Women — Obesity (G2: >50% CMI)**

Initial multivariable model ($R^2 = 0.29$):

**Cytokine**	**B**	**95% CI (lower)**	**95% CI (upper)**	**p-value**
IL-1β	0.0421	-0.1028	0.1871	0.5317
IL-6	0.2067	-0.2102	0.6236	0.2951
IL-10	-0.0732	-0.4477	0.3014	0.6727
IFN-γ	-0.1249	-0.4527	0.2029	0.4157
TNF-α	-0.1088	-0.4848	0.2672	0.5336

Backward elimination steps (β shown from initial model):

**Step**	**Removed cytokine**	**β (initial)**	**95% CI (lower)**	**95% CI (upper)**	**p at removal**	**R^2 after removal**
1	IL-10	-0.0732	-0.4477	0.3014	0.6727	0.277
2	IL-1β	0.0421	-0.1028	0.1871	0.606	0.258
3	TNF-α	-0.1088	-0.4848	0.2672	0.5497	0.235
4	IL-6	0.2067	-0.2102	0.6236	0.1757	0.114

Final model:

Cytokine	B	p-value
IFN-γ	-0.1258	0.2003

Final fit: $R^2 = 0.114$; $F = 1.807$; $p(F) = 0.200262$
